# Endothelial cells and pulmonary arterial hypertension: apoptosis, proliferation, interaction and transdifferentiation

**DOI:** 10.1186/1465-9921-10-95

**Published:** 2009-10-13

**Authors:** Seiichiro Sakao, Koichiro Tatsumi, Norbert F Voelkel

**Affiliations:** 1 Department of Respirology (B2), Graduate School of Medicine, Chiba University, 1-8-1 Inohana, Chuo-ku, Chiba 260-8670, Japan; 2 Victoria Johnson Center for Obstructive Lung Diseases and Pulmonary and Critical Care Medicine Division, Virginia Commonwealth University, 1101 East Marshall Street, Sanger Hall, Richmond, Virginia 23298-0565, USA

## Abstract

Severe pulmonary arterial hypertension, whether idiopathic or secondary, is characterized by structural alterations of microscopically small pulmonary arterioles. The vascular lesions in this group of pulmonary hypertensive diseases show actively proliferating endothelial cells without evidence of apoptosis. In this article, we review pathogenetic concepts of severe pulmonary arterial hypertension and explain the term "complex vascular lesion ", commonly named "plexiform lesion", with endothelial cell dysfunction, i.e., apoptosis, proliferation, interaction with smooth muscle cells and transdifferentiation.

## Introduction

Severe pulmonary arterial hypertension (PAH), whether idiopathic or associated with known causes (secondary forms), may have a reversible component in a minority of the patients [1,2], but most patients with severe PAH at the time of their diagnosis have persistent structural alterations of their microscopically small pulmonary arterioles, i.e., pulmonary vascular remodeling believed to be caused by angiogenic proliferation of endothelial cells (EC) [3-6]. Complex pulmonary vascular lesions at sites of bifurcations that are often glomeruloid appearing and lumen obliterating, including the so-called plexiform lesions, are frequently found in the lungs of patients with severe PAH, including the lungs from patients with Eisenmenger physiology where the lung vessels are subjected to increased (shunt) blood flow [7]. Whether these complex vascular lesions can fully explain the PAH remains controversial.

In this article, we review pathogenetic concepts of severe PAH and explain the term "complex vascular lesion," commonly named "plexiform lesion," with EC dysfunction, i.e., apoptosis, proliferation, interaction with smooth muscle cells (SMC) and transdifferentiation.

## Initial EC apoptosis is followed by the emergence of apoptosis-resistant proliferating EC

Discordant stimulation of EC or an uncontrolled EC response are common events in many pathologic processes including atherosclerosis, allograft vasculopathy, hypertension, congestive heart failure, sepsis and inflammatory syndromes, and PAH [8]. These diseases have in common endothelial injury, which can result in EC apoptosis, dysfunction and activation [8].

Especially pulmonary endothelial injury caused by toxins [9], reactive oxygen species [10,11], autoimmune mechanisms [5], and shear stress[12,13] likely leads to severe PAH.

A recent study showed that bone morphogenic proteins (BMP) signaling reduced apoptosis of cultured pulmonary artery EC under conditions of serum deprivation and maintained the survival of cultured circulating endothelial progenitors from normal individuals but not from IPAH patients. These results support the hypothesis that loss-of-function mutations in the bone morphogenic protein receptor II (BMPRII) could lead to increased pulmonary EC apoptosis, representing a possible initiating mechanism in the pathogenesis of PAH [14].

Taraseviciene-Stewart et al recently described that blockade of EC growth factor receptors resulted in the potentiation of PAH and marked worsening of the pathological vascular remodeling, even reproducing some of the "angioproliferative" features typical of advanced PAH and this effect was reversed by inhibitors of apoptosis, suggesting that increased apoptosis of EC in response to loss of survival signaling created conditions favoring the emergence of apoptosis-resistant cells with increased growth potential [15]. Moreover, Campbell et al and Zhao et al have shown that overexpression of EC growth and survival factors, such as vascular endothelial growth factor (VEGF) and angiopoietin-1, prevented the development of monocrotaline-induced PAH [16,17], an effect that was associated with reduced EC apoptosis. Together, the findings suggest that EC growth and the emergence of phenotypically altered vascular cells in severe PAH is the consequence of initial apoptosis and subsequent selection of apoptosis-resistant, proliferative vascular cells. This concept is consistent with recent finding describing the absence of apoptotic cells in the plexiform lesions in the lungs from patients with severe PAH [12] as well as reduction of severe PAH in the rat model [15] by treatment with simvastatin, which induced apoptosis of the EC that had obliterated the pulmonary arterioles [18].

To study the dependence of exuberant EC proliferation on initial apoptosis, we adapted the CELLMAX artificial capillary system to analyze the effects of the VEGF receptor (VEGFR) I and VEGFR II antagonist (SU5416) on human pulmonary microvascular EC (HPMVEC) under conditions of pulsatile shear stress [19].

**Figure 1:**
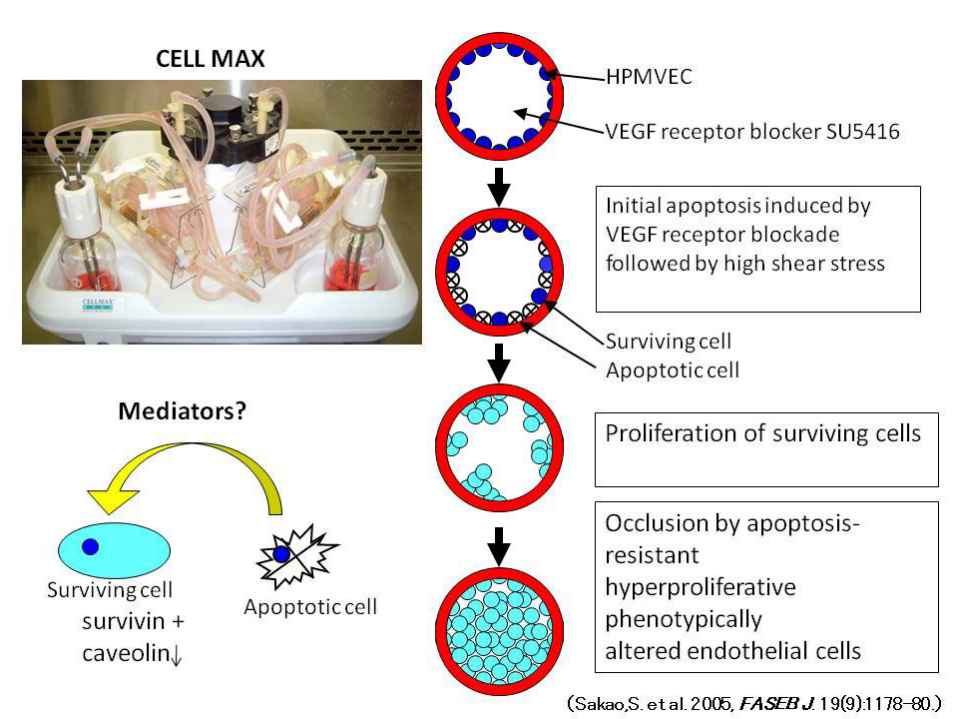
**The CELLMAX artificial capillary modules and sequence of events that leads from initial apoptosis to proliferation of apoptosis-resistant endothelial cells**. The combination of initial apoptosis induced by VEGF receptor blockade and high fluid shear stress generates apoptosis-resistant proliferative endothelial cells. *Definition of abbreviations*: HPMVEC = human pulmonary microvascular endothelial cell; VEGF = vascular endothelial growth factor; SU5416 = a combined VEGF I and II receptor blocker.

The experiments with human pulmonary microvascular EC (HPMVEC) seeded in the artificial capillary system demonstrated that a combined VEGF I and II receptor blocker (SU5416) induces EC apoptosis [19]. When this VEGF receptor blockade-induced apoptosis was followed by high fluid shear stress a hyperproliferative state was generated, and within 7 days phenotypically altered EC emerged [19]. These altered EC expressed the tumor marker survivin and the antiapoptotic protein Bcl-_XL _and were resistant to induction of apoptosis after challenge with TNF-α plus cycloheximide or hydrogen peroxide; in addition, the cells demonstrated survival in serum-free culture medium (Figure 1) [19].

Taken together our data reflect the paradox that growth factor-inhibition fosters the emergence of apoptosis-resistant and hyperproliferative cells [19]. This paradox has recently been described by Golpon et al [20] in experiments which resulted in the conclusion that there is "life after corpse engulfment". In these experiments it was shown that cells with apoptosis induced by UV irradiation, after they had been phagocytosed by other cells, released growth factors into the culture medium and that this conditioned medium made naïve epithelial- or endothelial cells apoptosis-resistant [20].

Whether in our shear stress experiments the SU5416 treated apoptotic cells were phagocytosed by neighboring cells of the CELLMAX system was not examined. In principle most cell types (not only professional phagocytes like macrophages) have the ability to phagocytose apoptosed cells [21-24] and we consider this possibility. It is unclear why the VEGF receptor blockade does not induce apoptosis in all of the EC and whether the surviving cells do so because they respond to survival signals which may be released by the dying cells. Alternatively or additionally it is conceivable that the EC contain some apoptosis-resistant precursor cells which expand under the conditions of our experiments [19]. Because VEGF receptor *inhibition *allows apoptosis-resistant EC growth and because Partovian et al showed that adenovirus-mediated VEGF over-expression reduced pulmonary hypertension [25] it is not clear that VEGF causes the angiogenic growth of the lumen-obliterating EC. It is possible that over-expression of the VEGF and VEGFR II proteins in the human pulmonary vascular lesions is a reflection of a vascular repair attempt. Again, the presence of VEGF and VEGFR II in the vascular lesions does not necessarily mean that VEGF actually causes the growth of the phenotypically altered and apoptosis-resistant cells.

Consistent with the result in this *in vitro *experiment, Masri and colleagues have reported *ex vivo *that pulmonary artery EC (PAECs) isolated from patients with idiopathic PAH (IPAH) exhibit an unusual hyperproliferative potential, with decreased susceptibility to apoptosis [26]. Together with accumulating evidence from previous studies [15,19,27], this study again provides support for the concept of an apoptosis-resistant and hyperproliferative EC in IPAH.

The above described *in vitro *experimental model appears to support the concept that apoptosis-resistant hyperproliferative EC can emerge at shear stress sensitive sites in the lung circulation in severe PAH. Although we do not address experimentally the factor or factors which confer apoptosis-resistance and phenotypical alterations of a subpopulation of endothelial stem-like cells, we suggest that blockade of the signal transduction of the obligatory EC survival factor, VEGF, in combination with high shear provide a selection pressure. The nature of the surviving and proliferating cells remains unclear. It is possible, as stated above, that the surviving and proliferating cells are precursor cells [28,29].

### Cross talk between endothelial and smooth muscle cells

The interactions of EC and SMC, which exist in the close contact of a functional syncytium, are involved in a process of new vessels formation that occurs during development, as part of wound repair, and during the reproductive cycle. One basic component of this interaction is the endothelial-induced recruitment, proliferation and subsequent differentiation of SMC [30-32].

Moreover, it was shown in *in vitro *studies that several growth factors or cytokines, such as activated transforming growth factor-β_1 _(TGF-β_1_) and IL-1β, had been produced by the EC and SMC in coculture and they might be involved in some of the effects exerted by the coculture on these cells [31,33,34]. TGF-β_1 _is a growth factor which is a potent stimulant of extracellular matrix synthesis and inhibits matrix degradation [35]. TGF-β_1 _has been shown to potentiate the development of intimal hyperplasia in animal models following arterial injury [36]. Thus, TGF-β_1 _appears to be an important mediator of the increased extracellular matrix deposition which occurs during vascular wall remodeling. IL-1β is one of inflammatory cytokines and its elevated serum levels in PAH have been reported [37].

Theories concerning the detailed pathobiology of PAH have focused on factors produced by EC and SMC and their response to different mediators. Prostacyclin (PGI_2_), a protein produced by EC and whose known target is SMC, could be one of the vasodilators. In patients with PAH, the levels of PGI_2 _are reduced [38]. Prostacyclin modulates the vasodilator response of SMC in the case of acute hypoxia [39].

We have previously hypothesized that the development of severe angioproliferative PAH is associated with initial EC apoptosis followed by the emergence of apoptosis-resistant proliferating EC [19]. However, the precise control of the balance between pulmonary arterial SMC (PASMC) proliferation and apoptosis is important in maintaining the structural and functional integrity of the pulmonary vasculature. In severe angioproliferative PAH, this balance seems to be disturbed such that there is increased PASMC proliferation and decreased apoptosis, leading to vessel wall thickening and vascular remodeling, i.e., hyperplasia of PASMC [40-43]. Indeed, severe angioproliferative PAH is characterized by complex precapillary arteriolar lesions [7,44-46], which contain phenotypically altered endothelial and smooth muscle cells [7]. Interestingly acquisition of resistance to apoptosis and increased rates of proliferation of PASMC appear to be necessary for neointima formation [47-52]. This phenotype plasticity, the dedifferentiation of mature, nonproliferative PASMC into proliferative PASMC, is a process central to vascular remodeling [53,54].

**Figure 2:**
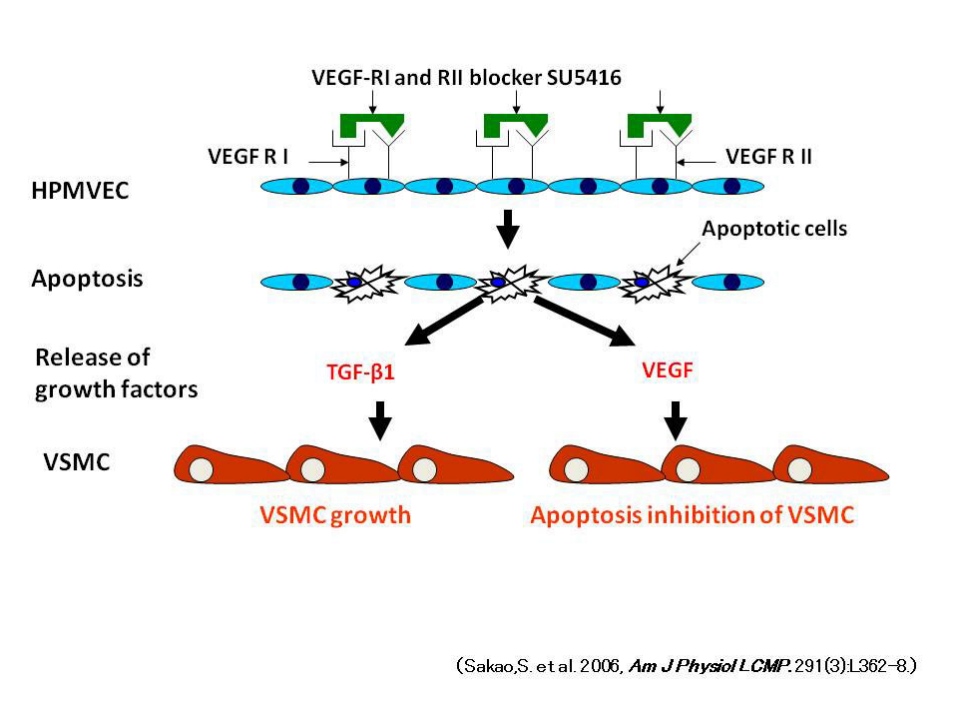
**Sequence of events that leads from SU-5416-induced VEGF blockade to the increased growth of VSMC**. VEGF receptor blockade induces apoptosis of vascular endothelial cells. Apoptotic endothelial cells release growth factors such as VEGF and TGF-β_1_, and, whereas VEGF inhibits apoptosis, TGF-β_1 _promotes VSMC proliferation. *Definition of abbreviations*: HPMVEC = human pulmonary microvascular endothelial cell; VSMC = vascular smooth muscle cell; TGF-β_1 _= transforming growth factor-β_1_; VEGF = vascular endothelial growth factor; SU5416 = a combined VEGF I and II receptor blocker.

We have previously demonstrated that EC death results in the selection of an apoptosis-resistant, proliferating and phenotypically altered EC phenotype [19]. Therefore we postulated that the initial apoptosis of EC induced the release of mediators which caused VSMC proliferation. To study this hypothesis, apoptosis of microvascular EC was induced by VEGF receptor blockade using the combined VEGFR-I and II blocker SU5416 and it was shown that serum-free medium conditioned by apoptosed EC caused proliferation of vascular SMC compared with serum-free medium conditioned by non-apoptosed EC [55]. It was also shown that serum-free medium conditioned by apoptosed EC is characterized by increased concentrations of TGF-β_1 _and VEGF compared with serum-free medium conditioned by non-apoptosed EC, and that TGF-β_1 _blockade prevented the proliferation of cultured vascular SMC [55]. In conclusion, EC death induced by VEGF receptor blockade leads to the production of factors, in particular TGF-β_1_, which activates vascular SMC proliferation, i.e., that EC apoptosis may stimulate vascular SMC growth (Figure 2) [55].

Moreover, several recent studies showed that EC seeding of injured arterial wall segments appears to limit the SMC response to injury. It was shown that EC seeding of endarterectomized canine arteries decreased the intimal hyperplastic response [56] and that EC seeding of injured hypercholesterolemic rabbit femoral arteries also limits the intimal hyperplastic response [57]. It is, therefore, reasonable to hypothesize that apoptosed EC may lose their control over SMC allowing SMC growth.

Recent studies suggest that, in response to intimal injury, synthetic/proliferative SMC migrated to the intima can generate proinflammatory molecules to promote WBC infiltration of the artery wall [53,58,59]. EC injury caused by proinflammatory molecules may lead to EC apoptosis and SMC growth and thus a EC apoptosis-SMC growth loop could result in the progression of PAH.

It is likely that dysregulated growth factors or cytokines produced by EC and SMC exert autocrine or paracrine effects which contribute to the progression of remodeling in pulmonary artery that results in PAH.

### Endothelial-Mesenchymal transdifferentiation

Transdifferentiation is a form of metaplasia and the conversion of one differentiated cell type into another, with or without intervening cell division, so this mechanism challenges the preconceived ideas that the terminal differentiated state is fixed. Indeed, it is now generally accepted that "differentiation" can sometimes be reversed or altered [60].

In the neointima formation and vascular remodeling fibroblasts in the pulmonary vascular wall play specific roles in the response to injury, including rapid migration, proliferation, synthesis of connective tissue, contraction, cytokine production, and, most importantly, transdifferentiation into other types of cells (e.g., PASMC) [61].

Hypoxia-induced changes in fibroblasts' proliferative and matrix-producing phenotypes are accompanied by the appearance of smooth muscle α-actin in tissues from pulmonary hypertensive subjects, suggesting that some of the fibroblasts transdifferentiate into myofibroblasts [62]. This transdifferentiation involves a complex network of microenvironmental factors and pathways in which extracellular matrix components as well as growth factors, cytokines, and adhesion molecules may play a role [63].

The intriguing possibility that intimal SMC may arise from the endothelium has received some attention [64,65]. In the systemic circulation, Arciniegas et al showed that mesenchymal cells that contribute to the intimal thickening may arise from the endothelium by using *in vivo *and *in vitro *methods [66].

Severe angioproliferative PAH is characterized by complex pulmonary precapillary arteriolar lesions [7,44-46], which contain phenotypically altered SMC and EC [7]. In addition to lumen-obliterating cell aggregates, which form the so-called plexiform lesions, muscularized arteries are also frequently present. Vasoconstriction as well as peptide (endothelin and angiotensin II) and nonpeptide (serotonin) growth factors have been postulated to be responsible for the muscularization of the pulmonary arteries in severe PAH [67-69]. Indeed "transitional cells" demonstrating features of both EC and vascular SMC have been identified in the plexiform lesions in the lungs from patients with severe angioproliferative PAH [70]. We hypothesize that an additional or alternative mechanism contributing to the muscularization of the pulmonary arteries may be transdifferentiation of pulmonary EC to mesenchymal cells.

**Figure 3:**
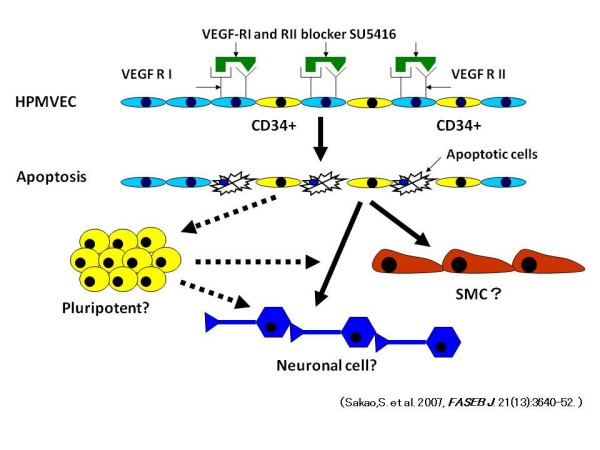
**Sequence of events in HPMVEC that lead from VEGF blockade by SU5416 to transdifferentiation to smooth muscle-like cells**. Endothelial cell death induced by VEGF receptor blockade and subsequent selection of progenitor-like cells leads to transdifferentiation to smooth muscle-like cells and neuronal cell. Dotted arrows mean hypothetical sequences of events. *Definition of abbreviations*: HPMVEC = human pulmonary microvascular endothelial cell; VSMC = vascular smooth muscle cell; SU5416 = a combined VEGF I and II receptor blocker.

To examine this hypothesis, we incubated HPMVEC with SU5416 and analyzed these cells utilizing quantitative-PCR, immunofluorescent staining and flow cytometry analysis [71]. *In vitro *studies of HPMVEC demonstrated that SU5416 suppressed PGI_2_S gene expression while potently inducing COX-2, VEGF and TGF-β_1 _expression, causing transdifferentiation of mature vascular EC (defined by Dil-ac-LDL, Lectin and Factor VIII) into SM-like (as defined by expression of α-SM actin) "transitional" cells, which coexpressed both endothelial and SM markers [71]. In this experiment, the SU5416-induced transdifferentiation was independent of TGF-β_1 _[71]. Although TGF-β_1 _was shown to be involved in inducing endothelial-mesenchymal transdifferentiation [72] and is known to promote SM-actin expression in nonmuscle cells (EC and fibroblasts derived from various tissues) [73,74], TGF-β_1 _is currently thought to be insufficient to induce expression of late SM differentiation marker SM myosin heavy chain (SM-MHC) in non-SMC lineage cells [74]. SU5416 expanded the number of CD34 and/or c-kit positive cells and caused transdifferentiation of CD34^+ ^cells, but not CD34^- ^cells. In conclusion, this data showed that SU5416 generated a selection pressure that killed some EC and expanded resident progenitor-like cells to transdifferentiate into SM like cells (Figure 3) [71]. Further, we fully realize the limitation of our data interpretation which is based on *in vitro *studies of cultured cells. However, we believe that our data may be consistent with the concept that transdifferentiation of pulmonary EC to mesenchymal cells may contribute to the muscularization of the pulmonary arteries.

The prevailing theory of the vascular SMC contribution to vascular lesions is that in pathological states, like atherosclerosis, SMCs migrate to the intima from the media of the vessel [75]. This concept, however, has been challenged by results derived from models of vascular injury, transplant arteriosclerosis, and human allograft studies, which all indicate that a portion of the cells bearing SMC differentiation markers in intimal lesions may have originated from the hematopoietic system and/or circulating progenitor cells [76-78]. Furthermore, a recent study demonstrated that smooth muscle progenitors were present in circulating blood [79], although the origin of these cells remains unknown. Concomitantly, it was shown that ~ 60% of SMC in atherosclerotic lesions of vein grafts were derived from the donor vessel wall and 40% from the recipient, possibly from circulating blood cells [80,81]. In the aggregate these reports strongly suggest the possibility of stem or progenitor cells as a source of SMC accumulation in atherosclerotic lesions. However, not all of the SMC within intimal lesions may be derived from bone marrow cells. Recently it was shown that, in addition to circulating progenitor cells, Sca-1^+ ^progenitor cells that reside in the adventitia can transdifferentiate into SMC-like neointimal cells [82], suggesting that not only bone marrow cells but also resident vessel wall precursor cells could exist and serve as a source of SMC to form neointimal lesions.

Ingram and colleagues [29] have resolved progenitor cells within a population of EC isolated from conduit vessels in the systemic circulation. These findings suggest that EC isolated from the vessel wall are enriched with progenitor cells that rapidly proliferate and can renew the entire population. This report confirms the unexpected finding in our study [71] that there is the presence of a small number of bone marrow-derived c-kit^+^, CD34^+ ^endothelial precursor cells among various batches of commercially available lung microvascular EC, suggesting the presence of such precursor cells in the adult lung.

The greater context of these findings, i.e., residential endothelial precursor cells and their transdifferentiation, may be a general mechanism for muscularization of vessels and, in the nondeveloping adult lung, a mechanism which participates in lung tissue homeostasis and repair of injured lung cells via utilization of resident lung tissue precursor cells.

### Genetic and/or epigenetic factors in PAH - a perspective

Genetic mutations, like BMPRII mutations that have been found in patients with familial and nonfamilial forms of IPAH [83], may contribute to cell growth control. Indeed, there is a growing literature that associates BMP and their receptors with cell growth control, even in cancers [84-86].

Not only somatic cell mutations may contribute to the hyperproliferative, apoptosis-resistant endothelium phenotype, but the unusual EC phenotype could also arise from a normal resident or itinerant lung cell population as a result of genomic events [71,87].

Not only "genetic", but also "epigenetic factors", should be considered as factors or conditions which induce the hyperproliferative, apoptosis-resistant endothelium phenotype. Epigenetics, here understood as a bridge between genotype and phenotype, can influence gene expression without changing the underlying DNA sequence, i.e., epigenetic modifications can express themselves via DNA methylation and histone modifications [88-91]. Dietary and hormonal influence can be envisioned to affect the pulmonary vessels in patients with IPAH, initiating or amplifying changes in the EC residing along the pulmonary vessels [92,93].

It is hypothesized that apoptosis-resistant, phenotypically altered and transdifferentiated EC may arise by genetic and epigenetic mechanisms.

## Conclusion

**Figure 4:**
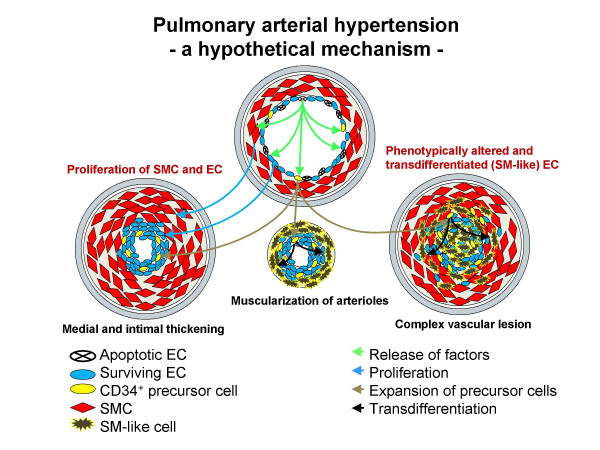
**A hypothetical mechanism of pulmonary arterial hypertension**. Sequence of events that leads from endothelial cell initial apoptosis to proliferation of apoptosis-resistant endothelial cells and vascular smooth muscle cells and endothlial-mesenchymal (SM-like) transdifferentiation. Apoptotic endothelial cells may release some kinds of factors that generate apoptosis-resistant proliferative endothelial cells, promote vascular smooth muscle cell proliferation and result in subsequent selection of progenitor-like cells leads to endothlial-mesenchymal (SM-like) transdifferentiation. These events may be a general mechanism for intimal and medial hypertrophy, muscularization of arterioles and complex vascular lesions. *Definition of abbreviations*: EC = endothelial cell; SMC = smooth muscle cell; SM-like = smooth muscle-like

It is tempting to speculate in the context of PAH that following EC apoptosis a selection of cells characterized by a high proliferative potential, including resident progenitor cells, results in a prevalence of hyperproliferative, apoptosis-resistant pulmonary vascular lesion cells that contribute to the irreversible and progressive nature which characterizes many forms of severe PAH (Figure 4).

## Competing interests

The authors declare that they have no competing interests.

## Authors' contributions

SS conceived of the report, contributed to its design and conception and drafted the manuscript. KT drafted the manuscript and contributed to its design and conception. NV contributed to its design and drafted the manuscript. All authors read and approved the final manuscript.

## References

[B1] Sitbon O Humbert M Nunes H Parent F Garcia G Herve P Rainisio M Simonneau G Long-term intravenous epoprostenol infusion in primary pulmonary hypertension: prognostic factors and survival J Am Coll Cardiol 2002 40 780 788 12204511 10.1016/s0735-1097(02)02012-0

[B2] Rimensberger PC Spahr-Schopfer I Berner M Jaeggi E Kalangos A Friedli B Beghetti M Inhaled nitric oxide versus aerosolized iloprost in secondary pulmonary hypertension in children with congenital heart disease: vasodilator capacity and cellular mechanisms Circulation 2001 103 544 548 11157720 10.1161/01.cir.103.4.544

[B3] Tuder RM Groves B Badesch DB Voelkel NF Exuberant endothelial cell growth and elements of inflammation are present in plexiform lesions of pulmonary hypertension Am J Pathol 1994 144 275 285 7508683 PMC1887146

[B4] Hirose S Hosoda Y Furuya S Otsuki T Ikeda E Expression of vascular endothelial growth factor and its receptors correlates closely with formation of the plexiform lesion in human pulmonary hypertension Pathol Int 2000 50 472 479 10886723 10.1046/j.1440-1827.2000.01068.x

[B5] Nicolls MR Taraseviciene-Stewart L Rai PR Badesch DB Voelkel NF Autoimmunity and pulmonary hypertension: a perspective Eur Respir J 2005 26 1110 1118 16319344 10.1183/09031936.05.00045705

[B6] Tuder RM Cool CD Yeager ME Taraseviciene-Stewart L Bull TM Voelkel NF The pathobiology of pulmonary hypertension Clin Chest Med 2001 22 405 418 11590837 10.1016/s0272-5231(05)70280-x

[B7] Cool CD Stewart JS Werahera P Miller GJ Williams RL Voelkel NF Tuder RM Three-dimensional reconstruction of pulmonary arteries in plexiform pulmonary hypertension using cell-specific markers. Evidence for a dynamic and heterogeneous process of pulmonary endothelial cell growth Am J Pathol 1999 155 411 419 10433934 10.1016/S0002-9440(10)65137-1 PMC1866857

[B8] Sumpio BE Riley JT Dardik A Cells in focus: endothelial cell Int J Biochem Cell Biol 2002 34 1508 1512 12379270 10.1016/s1357-2725(02)00075-4

[B9] Fishman AP Fishman MC Freeman BA Gimbrone MA Rabinovitch M Robinson D Gail DB Mechanisms of proliferative and obliterative vascular diseases: insights from the pulmonary and systemic circulations. NHLBI Workshop summary Am J Respir Crit Care Med 1998 158 670 674 9700149 10.1164/ajrccm.158.2.9803084

[B10] Ward JP Hypoxic pulmonary vasoconstriction is mediated by increased production of reactive oxygen species J Appl Physiol 2006 101 993 995 16675614 10.1152/japplphysiol.00480.2006

[B11] Weir EK Archer SL Counterpoint: Hypoxic pulmonary vasoconstriction is not mediated by increased production of reactive oxygen species J Appl Physiol 2006 101 995 998 16902070 10.1152/japplphysiol.00480a.2006

[B12] Ameshima S Golpon H Cool CD Chan D Vandivier RW Gardai SJ Wick M Nemenoff RA Geraci MW Voelkel NF Peroxisome proliferatoractivated receptor gamma (PPARgamma) expression is decreased in pulmonary hypertension and affects endothelial cell growth Circ Res 2003 92 1162 1169 12714563 10.1161/01.RES.0000073585.50092.14

[B13] Pi X Yan C Berk BC Big mitogen-activated protein kinase (BMK1)/ERK5 protects endothelial cells from apoptosis Circ Res 2004 94 362 369 14670836 10.1161/01.RES.0000112406.27800.6F

[B14] Teichert-Kuliszewska K Kutryk MJ Kuliszewski MA Karoubi G Courtman DW Zucco L Granton J Stewart DJ Bone morphogenetic protein receptor-2 signaling promotes pulmonary arterial endothelial cell survival: implications for loss-of-function mutations in the pathogenesis of pulmonary hypertension Circ Res 2006 98 209 217 16357305 10.1161/01.RES.0000200180.01710.e6

[B15] Taraseviciene-Stewart L Kasahara Y Alger L Hirth P Mc Mahon G Waltenberger J Voelkel NF Tuder RM Inhibition of the VEGF receptor 2 combined with chronic hypoxia causes cell death-dependent pulmonary endothelial cell proliferation and severe pulmonary hypertension FASEB J 2001 15 427 438 11156958 10.1096/fj.00-0343com

[B16] Campbell AI Zhao Y Sandhu R Stewart DJ Cell-based gene transfer of vascular endothelial growth factor attenuates monocrotaline-induced pulmonary hypertension Circulation 2001 104 2242 2248 11684638 10.1161/hc4201.097838

[B17] Zhao YD Campbell AI Robb M Ng D Stewart DJ Protective role of angiopoietin-1 in experimental pulmonary hypertension Circ Res 2003 92 984 991 12690034 10.1161/01.RES.0000070587.79937.F0

[B18] Taraseviciene-Stewart L Scerbavicius R Choe KH Cool C Wood K Tuder RM Burns N Kasper M Voelkel NF Simvastatin causes endothelial cell apoptosis and attenuates severe pulmonary hypertension Am J Physiol Lung Cell Mol Physiol 2006 291 L668 L676 16698853 10.1152/ajplung.00491.2005

[B19] Sakao S Taraseviciene-Stewart L Lee JD Wood K Cool CD Voelkel NF Initial apoptosis is followed by increased proliferation of apoptosis-resistant endothelial cells FASEB J 2005 19 1178 1180 15897232 10.1096/fj.04-3261fje

[B20] Golpon H Fadok V Taraseviciens-Stewart L Scerbavicius R Sauer C Welte T Henson PM Voelkel FN Life after corpse engulfment: Phagocytosis of apoptotic cells leads to VEGF secretion and cell growth FASEB J 2004 18 1716 1718 15345697 10.1096/fj.04-1853fje

[B21] Thompson CB Apoptosis in the pathogenesis and treatment of disease Science 1995 267 1456 7878464 10.1126/science.7878464

[B22] Henson PM Bratton DL Fadok VA Apoptotic cell removal Curr Biol 2001 11 R795 R805 11591341 10.1016/s0960-9822(01)00474-2

[B23] Fadok VA Bratton DL Henson PM Phagocyte receptors for apoptotic cells: recognition, uptake, and consequences J Clin Invest 2001 108 957 962 11581295 10.1172/JCI14122 PMC200959

[B24] Savill J Fadok V Corpse clearance defines the meaning of cell death Nature 2000 407 784 788 11048729 10.1038/35037722

[B25] Partovian C Adnot S Raffestin B Louzier V Levame M Mavier IM Lemarchand P Eddahibi S Adenovirus-mediated lung vascular endothelial growth factor overexpression protects against hypoxic pulmonary hypertension in rats Am J Respir Cell Mol Biol 2000 23 762 771 11104729 10.1165/ajrcmb.23.6.4106

[B26] Masri FA Xu W Comhair SA Asosingh K Koo M Vasanji A Drazba J Anand-Apte B Erzurum SC Hyperproliferative apoptosis-resistant endothelial cells in idiopathic pulmonary arterial hypertension Am J Physiol Lung Cell Mol Physiol 2007 293 L548 L554 17526595 10.1152/ajplung.00428.2006

[B27] Rai PR Cool CD King JAC Stevens T Burns N Winn RA Kasper M Voelkel NF The cancer paradigm of severe pulmonary arterial hypertension Am J Respir Crit Care Med 2008 178 558 564 18556624 10.1164/rccm.200709-1369PP PMC2542431

[B28] Ingram DA Mead LE Tanaka H Meade V Fenoglio A Mortell K Pollok K Ferkowicz MJ Gilley D Yoder MC Identification of a novel hierarchy of endothelial progenitor cells using human peripheral and umbilical cord blood Blood 2004 104 2752 2760 15226175 10.1182/blood-2004-04-1396

[B29] Ingram DA Mead LE Moore DB Woodard W Fenoglio A Yoder MC Vessel wall derived endothelial cells rapidly proliferate because they contain a complete hierarchy of endothelial progenitor cells Blood 2005 105 2783 2786 15585655 10.1182/blood-2004-08-3057

[B30] Lindahl P Johansson BR Levéen P Betsholtz C Pericyte loss and microaneurysm formation in PDGF-B-deficient mice Science 1997 277 242 245 9211853 10.1126/science.277.5323.242

[B31] Hirschi K Rohovsky SA D'Amore PA PDGF, TGF-β and heterotypic cell-cell interactions mediate the recruitment and differentiation of 10T1/2 cells to a smooth muscle cell fate J Cell Biol 1998 141 805 814 9566978 10.1083/jcb.141.3.805 PMC2132737

[B32] Hellström M Kalén M Lindahl P Abramsson A Betsholtz C Role of PDGF-B and PDGFR-β in recruitment of vascular smooth muscle cells and pericytes during embryonic blood vessel formation in the mouse Development 1999 126 3047 3055 10375497 10.1242/dev.126.14.3047

[B33] Antonelli-Orlidge A Saunders KB Smith SR D'Amore PA An activated form of transforming growth factor beta is produced by co-cultures of endothelial cells and pericytes Proc Natl Acad Sci USA 1989 86 4544 4548 2734305 10.1073/pnas.86.12.4544 PMC287307

[B34] Asakawa H Kobayashi T The effect of co-culture with human smooth muscle cells on the proliferation, the IL-1 beta secretion, the PDGF production and tube formation of human aortic endothelial cells Cell Biochem Funct 1999 17 123 130 10377958 10.1002/(SICI)1099-0844(199906)17:2<123::AID-CBF817>3.0.CO;2-3

[B35] Penttinen RP Kobayashi S Bornstein P Transforming growth factor-β increases mRNA for matrix proteins both in the presence and in the absence of changes in mRNA stability Proc Natl Acad Sci USA 1988 85 1105 1108 3422482 10.1073/pnas.85.4.1105 PMC279714

[B36] Majesky MW Lindner V Twardzik DR Production of transforming growth factor β_1_ J Clin Invest 1991 88 904 910 1832175 10.1172/JCI115393 PMC295478

[B37] Humbert M Monti G Brenot F Sitbon O Portier A Grangeot-Keros L Duroux P Galanaud P Simonneau G Emilie D Increased interleukin-1 and interleukin-6 serum concentrations in severe primary pulmonary hypertension Am J Respir Crit Care Med 1995 151 1628 1631 7735624 10.1164/ajrccm.151.5.7735624

[B38] Christman BW McPherson CD Newman JH King GA Bernard GR Groves BM Loyd JE An imbalance between the excretion of thromboxane and prostacyclin metabolites in pulmonary hypertension N Engl J Med 1992 327 70 75 1603138 10.1056/NEJM199207093270202

[B39] Mikhail G Chester AH Gibbs SR Borland JAA Banner NR Yacoub MH Role of vasoactive mediators in primary and secondary pulmonary hypertension Am J Cardiol 1998 82 254 255 9678304 10.1016/s0002-9149(98)00296-3

[B40] Rabinovitch M Elastase and the pathobiology of unexplained pulmonary hypertension Chest 1998 114 213 224 10.1378/chest.114.3_supplement.213s 9741572

[B41] Rubin LJ Cellular and molecular mechanisms responsible for the pathogenesis of primary pulmonary hypertension Pediatr Pulmonol Suppl 1999 18 194 197 10093141

[B42] Wagenvoort CA Wagenvoort N Primary pulmonary hypertension. A pathologic study of the lung vessels in 156 clinically diagnosed cases Circulation 1970 42 1163 1171

[B43] Wohrley JD Frid MG Moiseeva EP Orton EC Belknap JK Stenmark KR Hypoxia selectively induces proliferation in a specific subpopulation of smooth muscle cells in the bovine neonatal pulmonary arterial media J Clin Invest 1995 96 273 281 7615796 10.1172/JCI118031 PMC185198

[B44] Golpon HA Geraci MW Moore MD Miller HL Miller GJ Tuder RM Voelkel NF HOX genes in human lung: altered expression in primary pulmonary hypertension and emphysema Am J Pathol 2001 158 955 966 11238043 10.1016/S0002-9440(10)64042-4 PMC1850338

[B45] Tuder RM Chacon M Alger L Wang J Taraseviciene-Stewart L Kasahara Y Cool CD Bishop AE Geraci M Semenza GL Yacoub M Polak JM Voelkel NF Expression of angiogenesis-related molecules in plexiform lesions in severe pulmonary hypertension: evidence for a process of disordered angiogenesis J Pathol 2001 195 367 374 11673836 10.1002/path.953

[B46] Tuder RM Cool CD Geraci MW Wang J Abman SH Wright L Badesch D Voelkel NF Prostacyclin synthase expression is decreased in lungs from patients with severe pulmonary hypertension Am J Respir Crit Care Med 1999 159 1925 1932 10351941 10.1164/ajrccm.159.6.9804054

[B47] Diez J Fortuno M Zalba G Etayo J Fortuno A Ravassa S Beaumont J Altered regulation of smooth muscle cell proliferation and apoptosis in small arteries of spontaneously hypertensive rats Eur Heart J 1998 19 G29 G33 9717053

[B48] Guevara N Kim H Antonova E Chan L The absence of p53 accelerates atherosclerosis by increasing cell proliferation in vivo Nat Med 1999 5 335 339 10086392 10.1038/6585

[B49] Malik N Francis S Holt C Gunn J Thomas G Shepherd L Chamberlain J Newman C Cumberland D Crossman D Apoptosis and cell proliferation after porcine coronary angioplasty Circulation 1998 98 1657 1665 9778332 10.1161/01.cir.98.16.1657

[B50] Pollman M Hall J Mann M Zhang L Gibbons G Inhibition of neointimal cell bcl-x expression induces apoptosis and regression of vascular disease Nat Med 1998 4 222 227 9461197 10.1038/nm0298-222

[B51] Sata M Perlman H Muruve D Silver M Ikebe M Libermann T Oettgen P Walsh K Fas ligand gene transfer to the vessel wall inhibits neointima formation and overrides the adenovirus-mediated T cell response Proc Natl Acad Sci USA 1998 95 1213 1217 9448311 10.1073/pnas.95.3.1213 PMC18722

[B52] Zhang S Fantozzi I Tigno DD Yi ES Platoshyn O Thistlethwaite PA Kriett JM Yung G Rubin LJ Yuan JX-J Bone morphogenetic proteins induce apoptosis in human pulmonary vascular smooth muscle cells Am J Physiol Lung Cell Mol Physiol 2003 285 L740 L754 12740218 10.1152/ajplung.00284.2002

[B53] Owens GK Kumar MS Wamhoff BR Molecular regulation of vascular smooth muscle cell differentiation in development and disease Physiol Rev 2004 84 767 801 15269336 10.1152/physrev.00041.2003

[B54] Li S Sims S Jiao Y Chow LH Pickering JG Evidence from a novel human cell clone that adult vascular smooth muscle cells can convert reversibly between noncontractile and contractile phenotypes Circ Res 1999 85 338 348 10455062 10.1161/01.res.85.4.338

[B55] Sakao S Taraseviciene-Stewart L Wood K Cool CD Voelkel NF Apoptosis of pulmonary microvascular endothelial cells stimulates vascular smooth muscle cell growth Am J Physiol Lung Cell Mol Physiol 2006 291 L362 L368 16617095 10.1152/ajplung.00111.2005

[B56] Bush HJ Jakubowski JA Sentissi JM Neointimal hyperplasia occurring after carotid endarterectomy in a canine model: Effect of endothelial cell seeding vs perioperative aspirin J Vasc Surg 1987 5 118 125 3795378

[B57] Conte MS Endothelial cell resurfacing improves remodeling of balloon-injured arteries in the hypercholesterolemic rabbit Surg Forum 1996 47 333 336

[B58] Rainger GE Nash GB Cellular pathology of atherosclerosis: smooth muscle cells prime cocultured endothelial cells for enhanced leukocyte adhesion Circ Res 2001 88 615 622 11282896 10.1161/01.res.88.6.615

[B59] Zeiffer U Schober A Lietz M Liehn EA Erl W Emans N Yan ZQ Weber C Neointimal smooth muscle cells display a proinflammatory phenotype resulting in increased leukocyte recruitment mediated by P-selectin and chemokines Circ Res 2004 94 776 784 14963004 10.1161/01.RES.0000121105.72718.5C

[B60] Tosh D Slack JM How cells change their phenotype Nature Reviews Molecular Cell Biology 2002 3 187 194 11994739 10.1038/nrm761

[B61] Sartore S Chiavegato A Faggin E Franch R Puato M Ausoni S Pauletto P Contribution of adventitial fibroblasts to neointima formation and vascular remodeling Circ Res 2001 89 1111 1121 11739275 10.1161/hh2401.100844

[B62] Stenmark KR Durmowicz AG Dempsey EC Bishop JE, Reeves JJ, Laurent GJ Modulation of vascular wall cell phenotype in pulmonary hypertension Pulmonary Vascular Remodeling 1995 Portland Press, London, UK

[B63] Sisbarro L Ihida-Stansbury K Stevens T Bauer N McMurtry I Jones PL The extracellular matrix microenvironment specifies pulmonary endothelial cell identity: roles of tenascin-C and RhoA Chest 2005 128 16373827 10.1378/chest.128.6_suppl.564S

[B64] Majesky MW Schwartz SM An origin for smooth muscle cells from endothelium? Circ Res 1997 80 601 603 9118492

[B65] Schwartz SM Perspectives series: cell adhesion in vascular biology. Smooth muscle migration in atherosclerosis and restenosis J Clin Invest 1997 99 2814 2817 9185501 10.1172/JCI119472 PMC508129

[B66] Arciniegas E Ponce L Hartt Y Graterol A Carlini RG Intimal thickening involves transdifferentiation of embryonic endothelial cells Anat Rec 2000 258 47 57 10603448 10.1002/(SICI)1097-0185(20000101)258:1<47::AID-AR6>3.0.CO;2-W

[B67] Zamora MR Stelzner TJ Webb S Panos RJ Ruff LJ Dempsey EC Overexpression of endothelin-1 and enhanced growth of pulmonary artery smooth muscle cells from fawn-hooded rats Am J Physiol Lung Cell Mol Physiol 1996 270 L101 L109 10.1152/ajplung.1996.270.1.L101 8772532

[B68] Okada K Bernstein M Zhang W Schuster D Botney M Angiotensin-converting enzyme inhibition delays pulmonary vascular neointimal formation Am J Respir Crit Care Med 1998 158 939 950 9731029 10.1164/ajrccm.158.3.9710007

[B69] Lee SL Wang WW Moore BJ Fanburg BL Dual effect of serotonin on growth of bovine pulmonary artery smooth muscle cells in culture Circ Res 1991 68 1362 1368 1850332 10.1161/01.res.68.5.1362

[B70] Cool CD Wood K Voelkel NF Transdifferentiation of endothelial cells in primary pulmonary hypertension Am J Resp Crit Care Med 2004 167 A844

[B71] Sakao S Taraseviciene-Stewart L Cool CD Tada Y Kasahara Y Kurosu K Tanabe N Takiguchi Y Tatsumi K Kuriyama T Voelkel NF VEGF-R blockade causes endothelial cell apoptosis, expansion of surviving CD34+ precursor cells and transdifferentiation to smooth muscle-like and neuronal-like cells FASEB J 2007 21 3640 3652 17567571 10.1096/fj.07-8432com

[B72] Frid MG Kale VA Stenmark KR Mature vascular endothelium can give rise to smooth muscle cells via endothelial-mesenchymal transdifferentiation: in vitro analysis Circ Res 2002 14 1189 1196 10.1161/01.res.0000021432.70309.28 12065322

[B73] Arciniegas E Sutton AB Allen TD Schor AM Transforming growth factor beta 1 promotes the differentiation of endothelial cells into smooth muscle-like cells in vitro J Cell Sci 1992 103 521 529 1478952 10.1242/jcs.103.2.521

[B74] Hautmann MB Adam PJ Owens GK Similarities and differences in smooth muscle -actin induction by TGF-s in smooth muscle versus non-smooth muscle cells Arterioscler Thromb Vasc Biol 1999 19 2049 2058 10479645 10.1161/01.atv.19.9.2049

[B75] Ross R Glomset JA Atherosclerosis and the arterial smooth muscle cell: proliferation of smooth muscle is a key event in the genesis of the lesions of atherosclerosis Science 1973 180 1332 1339 4350926 10.1126/science.180.4093.1332

[B76] Sata M Saiura A Kunisato A Tojo A Okada S Tokuhisa T Hirai H Makuuchi M Hirata Y Nagai R Hematopoietic stem cells differentiate into vascular cells that participate in the pathogenesis of atherosclerosis Nat Med 2002 8 403 409 11927948 10.1038/nm0402-403

[B77] Shimizu K Sugiyama S Aikawa M Fukumoto Y Rabkin E Libby P Mitchell RN Host bone-marrow cells are a source of donor intimal smooth-muscle-like cells in murine aortic transplant arteriopathy Nat Med 2001 7 738 741 11385513 10.1038/89121

[B78] Glaser R Lu MM Narula N Epstein JA Smooth muscle cells, but not myocytes, of host origin in transplanted human hearts Circulation 2002 106 17 19 12093763 10.1161/01.cir.0000021923.58307.8f

[B79] Simper D Stalboerger PG Panetta CJ Wang S Caplice NM Smooth muscle progenitor cells in human blood Circulation 2002 106 1199 1204 12208793 10.1161/01.cir.0000031525.61826.a8

[B80] Hu Y Davison F Ludewig B Erdel M Mayr M Url M Dietrich H Xu Q mooth muscle cells in transplant atherosclerotic lesions are originated from recipients, but not bone marrow progenitor cells Circulation 2002 106 S1834 1839 10.1161/01.cir.0000031333.86845.dd 12356638

[B81] Hu Y Mayr M Metzler B Erdel M Davison F Xu Q Both donor and recipient origins of smooth muscle cells in vein graft atherosclerotic lesions Circ Res 2002 91 e13 e20 12364395 10.1161/01.res.0000037090.34760.ee

[B82] Hu Y Zhang Z Torsney E Afzal AR Davison F Metzler B Xu Q Abundant progenitor cells in the adventitia contribute to atherosclerosis of vein grafts in ApoE-deficient mice J Clin Invest 2004 113 1258 1265 15124016 10.1172/JCI19628 PMC398426

[B83] Aldred MA Vijayakrishnan J James V Soubrier F Gomez-Sanchez MA Martensson G Galie N Manes A Corris P Simonneau G Humbert M Morrell NW Trembath RC BMPR2 gene rearrangements account for a significant proportion of mutations in familial and idiopathic pulmonary arterial hypertension Hum Mutat 2006 27 212 213 16429403 10.1002/humu.9398

[B84] Beck SE Jung BH Del Rosario E Gomez J Carethers JM BMP induced growth suppression in colon cancer cells is mediated by p21WAF1 stabilization and modulated by RAS/ERK Cell Signal 2007 19 1465 1472 17317101 10.1016/j.cellsig.2007.01.017 PMC3444522

[B85] Katoh M Networking of WNT, FGF, Notch, BMP, and Hedgehog signaling pathways during carcinogenesis Stem Cell Rev 2007 3 30 38 17873379 10.1007/s12015-007-0006-6

[B86] Ye L Lewis-Russell JM Kyanaston HG Jiang WG Bone morphogenetic proteins and their receptor signaling in prostate cancer Histol Histopathol 2007 22 1129 1147 17616940 10.14670/HH-22.1129

[B87] Stevens T Gillespie MN The hyperproliferative endothelial cell phenotype in idiopathic pulmonary arterial hypertension Am J Physiol Lung Cell Mol Physiol 2007 293 L546 L547 17601794 10.1152/ajplung.00246.2007

[B88] Bernstein BE Meissner A Lander ES The mammalian epigenome Cell 2007 128 669 861 17320505 10.1016/j.cell.2007.01.033

[B89] Goldberg AD Allis CD Bernstein E Epigenetics: a landscape takes shape Cell 2007 128 635 638 17320500 10.1016/j.cell.2007.02.006

[B90] Grewal SI Moazed D Heterochromatin and epigenetic control of gene expression Science 2003 301 798 802 12907790 10.1126/science.1086887

[B91] Groth A Rocha W Verreault A Almouzni G Chromatin challenges during DNA replication and repair Cell 2007 128 721 733 17320509 10.1016/j.cell.2007.01.030

[B92] Taraseviciute A Voelkel NF Severe pulmonary hypertension in postmenopausal obese women Eur J Med Res 2006 11 198 202 16723293

[B93] Morse JH Horn EM Barst RJ Hormone replacement therapy: a possible risk factor in carriers of familial primary pulmonary hypertension Chest 1999 116 847 10492306 10.1378/chest.116.3.847

